# Addressing the gaps: a mixed-methods scoping review on comprehensive sexuality education training for school educators

**DOI:** 10.1136/bmjopen-2025-111342

**Published:** 2026-06-19

**Authors:** Amani Isa, Aisha Shalash, Mallak Ghanem, Maysaa Nemer

**Affiliations:** 1 Institute of Community and Public Health, Birzeit University, Birzeit, Palestine

**Keywords:** Adolescents, PUBLIC HEALTH, Schools, EDUCATION & TRAINING (see Medical Education & Training)

## Abstract

**Abstract:**

**Introduction:**

Comprehensive sexuality education is an educational approach that addresses the cognitive, emotional, physical and social aspects of sexuality. When taught consistently and age-appropriately, it expands survival, health and life benefits for young people everywhere. To provide an efficient, comprehensive sexuality education curriculum, educators must be equipped with proper training.

**Objective:**

This scoping review aimed to map and identify research gaps in the existing literature on educators’ training in comprehensive sexuality education, examine facilitators and barriers to training and curriculum implementation, and highlight areas requiring further investigation.

**Methods:**

A comprehensive search of six academic databases was conducted, followed by a mixed-methods approach to analyse the data. Narrative synthesis and data mapping were used to analyse study characteristics for all the included studies. Descriptive statistics were used to analyse quantitative data and thematic analysis guided by an inductive approach was used to analyse common themes and sub-themes for qualitative data.

**Results:**

A total of 74 studies (qualitative n=31; quantitative n=24; mixed methods n=19) were included in this review. The results of this review found that preservice educator training to be the most effective training approach. Additionally, results found that the best training duration is context-dependent but should incorporate a combination of in-person and online training methods that include follow-up technical support strategies.

**Conclusions:**

Due to the regional disparities identified in this review, future research needs to focus on the Eastern Mediterranean and Southeast Asia regions. The findings of this scoping review provide researchers with evidence-based knowledge to make practical recommendations for an efficient comprehensive sexuality education training programme for educators while addressing barriers to training, as well as facilitators and barriers to implementation.

**STRENGTHS AND LIMITATIONS OF THIS STUDY**

- The review was guided by a structured conceptual framework and the Arksey & O’Malley mixed-methods scoping review design.
- A comprehensive search of six databases was conducted, with date limitations applied to peer-reviewed studies to ensure the inclusion of current practices and technologies, while reference lists of included articles were hand-searched to ensure reliability and validity.
- A mixed-methods approach yielded a holistic view: quantitative statistics were complemented by qualitative insights from individual perspectives.
- Including global coverage enabled assessment of worldwide barriers and facilitators to comprehensive sexuality education training and implementation, providing clear directions for adaptation and classroom application.
- Limitations can be found by potential selection/publication bias due to the inclusion of only English and Arabic studies, based on languages spoken by the research team, as well as the exclusion of grey literature, which may have omitted relevant unpublished evidence.

## Introduction

 Comprehensive sexuality education (CSE) is an educational approach that consists of a range of topics, including the cognitive, emotional, physical and social aspects of sexuality.^1^ It is a lifelong process in which one learns about oneself and others as sexual beings from biological, psychological and sociocultural perspectives, and it plays an essential role in the life of an adolescent aged 10–19.^2^ CSE does not only talk about sexual behaviours, abstinence, family planning, childbirth and sexually transmitted infections (STIs). Instead, it is a comprehensive curriculum that refers to the ‘depth and consistency’ of topics.^3^ It discusses SE topics such as puberty, menstruation, family life and relationships, sexuality, human rights, sexual abuse and gender-based violence.^3^ When effectively implemented and paired with access to essential sexual and reproductive health (SRH) services, CSE enables young people to make informed choices about their relationships and sexuality.

Research shows that CSE needs to be taught in schools by properly trained educators because educator preparedness has been positively shown to increase programme effectiveness and deliver consistent and accurate information to students.^4 5^ Unfortunately, CSE curricula are inconsistently implemented in education systems across the globe and seldom adhere to formal international guidelines.^6^ Furthermore, research evidence supports the implementation of CSE curricula and negates the effectiveness of abstinence programmes as they do not increase protection for adolescents from the delay of initiation of sexual activity or decrease teenage pregnancy rates.^2 7 8^ When CSE is taught consistently and age appropriately, meaning it meets the cognitive and emotional maturity and needs of students, it expands survival, health and life benefits for young people worldwide.^9 10^ Students exposed to culturally and age-appropriate content at a young age are later more confident in discussing sexually related topics and are more likely to report instances of sexual abuse than students who did not receive CSE.^5^ CSE has also been found to play an important role in protecting children from abuse by teaching them about their bodies and promoting healthy practices.^11^

Educators, including school teachers, counsellors and nurses, serve as facilitators of learning, moral guides and role models.^12 13^ Despite parental opposition, increased social media exposure among adolescents has led parents to recognise that they cannot avoid topics that were once considered taboo, resulting in support of school-implemented CSE.^14 15^ However, it has been reported that there is insufficient training in SE topics during preservice education, which hinders the implementation of CSE in the classroom.^16 17^ Training and professional development are essential in enhancing educators’ abilities to engage students in learning, particularly in the context of CSE instruction.^18^ Furthermore, preservice training alone is insufficient and must be followed up with continuous education for in-service educators and should use in-person group training sessions that last less than 48 hours.^19^,^21^ Sociocultural factors, such as religion, gender norms and regional beliefs, have been shown to influence the implementation of CSE and shape the way stakeholders, including school administrators, religious leaders, policymakers and community elders, approach CSE globally.^22^

Many countries, especially low- and middle-income countries (LMICs) that have adopted faith-based education approaches, view the majority of CSE teachings as a Westernised ideology and lean towards abstinence-only programmes, limiting young people from a wide range of SRH education.^23 24^ However, barriers to CSE implementation are not exclusive to LMICs. High-income countries (HICs) and societies that are recognised as partially secular have been found to still be highly influenced by Judeo-Christian values, causing ideological debate over the implementation and programme designs of CSE.^25^ Over the past four decades, the US federal government has dedicated major funding to abstinence-only programmes, which do not include CSE.^26 27^ Educators from rural southern regions of the USA stated feeling constrained in what they could teach about sexuality because of stakeholder gatekeepers, which are individuals or groups that influence decision-making processes and shape policy outcomes.^28 29^ Gender has also been found to influence CSE implementation. Stakeholders in Saudi Arabia regarded CSE as essential, but gender considerations highly influenced their views towards CSE. They viewed boys’ SRH needs as needing protection from STIs and girls’ SRH needs as the preservation of their virginity and, thus, their honour.^25^ This perspective could be detrimental because, despite the different developmental trajectories of boys and girls, their SRH needs should not be determined by societal expectations.

While numerous studies exist on CSE content and student outcomes, few studies have focused on the impact of available training on educators’ outcomes, including their knowledge, attitudes and effectiveness.^30 31^ Limited research also exists in regions such as the Eastern Mediterranean (EMRO) and South-East Asia (SEARO).^32 33^ This limits the understanding of how to develop adaptable CSE training and implement culture-sensitive curricula. Additionally, inconsistent training content, methods and durations have made it challenging for programme development and implementation.^31 34^ This challenge emphasises the importance of developing standardised yet adaptable guidelines that can be tailored to meet the needs of specific regions, cultures or religions for effective CSE training and implementation.

By bridging these gaps, this scoping review provides a fundamental basis for effective CSE educator training. It will map the extent of existing knowledge to identify facilitators and barriers related to the training of educators in CSE programmes, including training methods, content and implementation challenges, to provide new evidence-based methods, facilitators and barriers for educator CSE training and classroom implementation. Additionally, it will aim to enhance the development of CSE educator training while focusing on the needs and perspectives of educators in socially and culturally sensitive environments.

### Methods

A mixed-methods approach guided by the Arksey and O’Malley framework was used to conduct this scoping review.^35^ Additionally, this scoping review used the Preferred Reporting Items for Systematic Reviews and Meta-Analysis Extension for Scoping Reviews (PRISMA) checklist as a guide for writing and can be found in online supplemental 1.^36^

### Identifying the research question

Since this study aimed to identify and map existing CSE training for educators worldwide, the population, concept, context framework was used.^37^ The framework was defined as follows:

- Population: school teachers, counsellors and nurses
- Concept: CSE training, SRH education and CSE curricula
- Context: schools and education systems

### Identifying relevant studies

Using key concepts from the identified research question, a search strategy was developed. To determine all relative search terms that were identified and used in database searches, database-specific subject headings like MeSH (Medical Subject Headings) terms in PubMed or thesaurus terms in other databases were examined. This helped to ensure that all synonyms and appropriate terms were used. The terms used in the search strategy were: ((comprehensive ((comprehensive sexual education) OR (sexual reproductive health)) AND (training) AND (teachers) OR (counselors) OR (school nurses)) AND (adolescents) OR (teen) OR (preteen) OR (youth)). The database search was conducted using the following six electronic databases: PubMed, Web of Science, Scopus, Embase, CINAHL and PsycINFO. Each database search strategy can be found in online supplemental file 2.

### Study selection process

#### Inclusion criteria

Peer-reviewed studies that explored specific CSE training programmes, interventions or campaigns for school teachers, counsellors or nurses in a school or education system were included. Both qualitative and quantitative studies were included. Only studies published during or after the year 2000 to keep up with emerging trends and studies published in the English or Arabic language were included.

#### Exclusion criteria

Studies that did not describe a specific CSE training programme, intervention or campaign were excluded. Studies that exclusively focused on populations other than school teachers, counsellors or nurses and were fully conducted somewhere other than a school or education system were not included. All books, commentaries, abstracts, posters, letters to editors, editorials or grey literature were also excluded. Additionally, studies published before the year 2000 or in a language other than English or Arabic were excluded.

#### Study screening

After completing the database searches in April 2024, all the studies were imported into EndNote, a reference management software used to manage and organise references,^38^ and then exported into Covidence, a web-based software used to conduct systematic and scoping reviews.^39^ These software programmes also work to detect and remove duplicate references. Four researchers (AI, AS, MG and MN) independently conducted title and abstract screening. The primary researcher, AI, screened all titles and abstracts, as well as full-text screening. To ensure consistency and accuracy, title and abstract screening, as well as full-text screening, were split among the three other researchers: AS, MG and MN. Conflicts were discussed, and if a consensus could not be reached, a third reviewer was consulted. All reference lists of the included studies were hand-searched to ensure that all studies were found in the database search.^35^ Citation searching was also conducted by examining the reference lists of included studies to identify additional relevant sources.^40^ Studies found during the hand and citation search were then imported into Covidence to undergo title and abstract as well as full text screening.

#### Data extraction

The primary researcher, AI, extracted and charted all the data from the included studies, and to further ensure validity and accuracy, AS extracted 10% of the relevant data. Data was initially extracted using a Covidence template, then exported into a Microsoft Excel spreadsheet.^41^ The following information was extracted for each included study: Covidence number, lead author, year of publication, journal, title, country, WHO region classification, aim(s), study design, study setting, study dates, population description, sample size, inclusion criteria, exclusion criteria, method of recruitment, intervention (description of the mentioned CSE programme), outcomes 1 (impact of CSE training on educators’ attitudes, perceptions, beliefs, changes in knowledge, skills and teaching practices), outcomes 2 (reported barriers of training and reported barriers and facilitators for CSE implementation), recommendations and conflicts or limitations.

#### Collating, analysing and reporting the results

Narrative synthesis and data mapping were used to analyse all the included studies collectively. All included studies were first summarised descriptively in Excel according to key study characteristics: study design, year of publication, WHO region (African (AFRO), Americas (AMRO/PAHO), EMRO, European (EURO), SEARO, Western Europe and Central Asia (WPRO)), country income status, target population, training method, training duration and recurring training programmes. Frequencies and percentages were calculated to describe the distribution of studies across these categories. Thematic analysis was conducted for data extracted from qualitative studies using MAXQDA.^42^ This analysis method used an inductive approach, guided by Braun and Clarke’s method, which allows themes to be derived directly from the data rather than being based on pre-existing frameworks.^43^ Findings are presented through tables to map the characteristics of the included studies and synthesise the main patterns across the evidence base.

## Results

**Figure 1:**
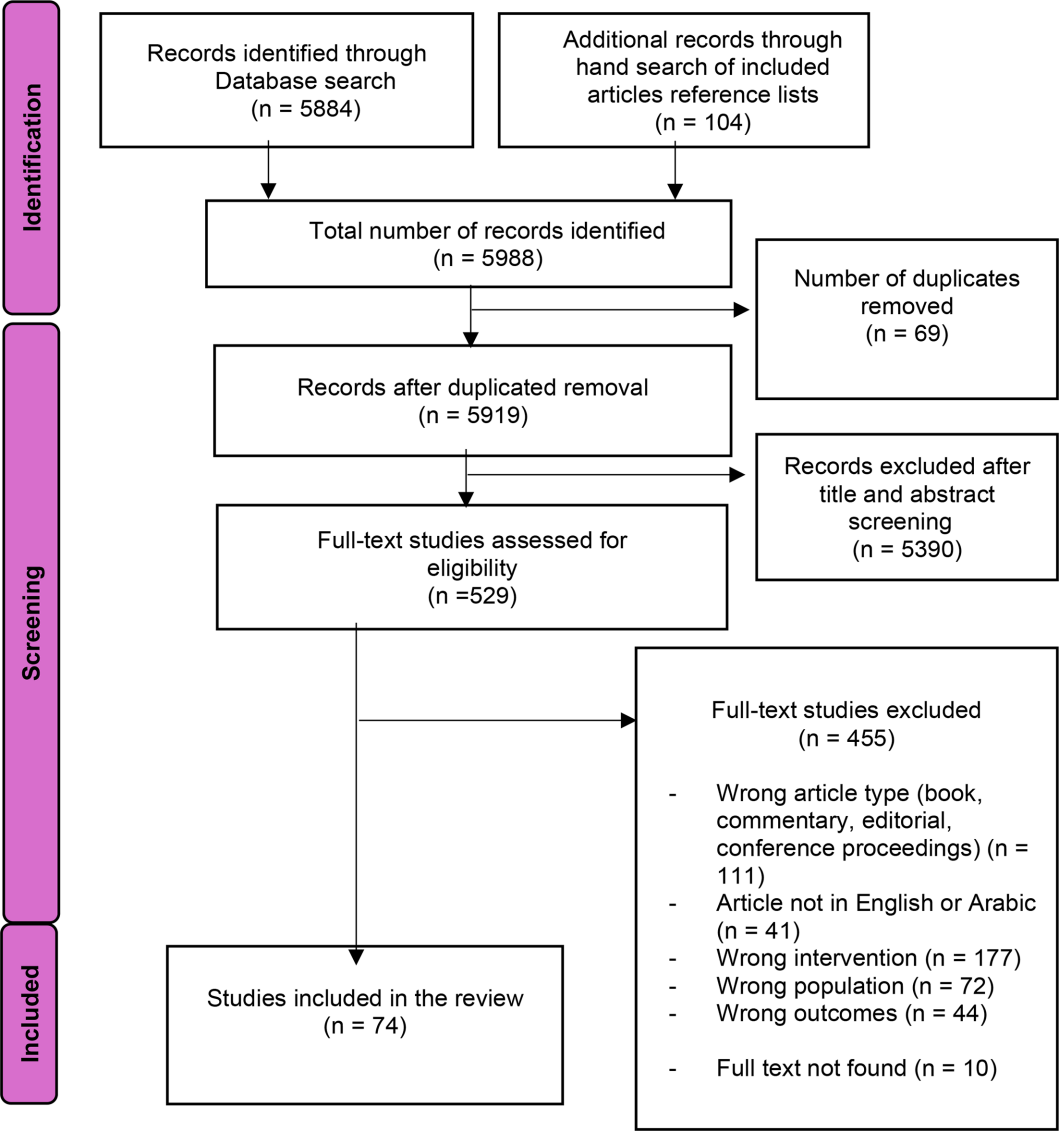
PRISMA flow diagram of the study selection process. This figure shows the process of identification, screening, eligibility assessment and inclusion of studies in the review. PRISMA, Preferred Reporting Items for Systematic Reviews and Meta-Analyses.

A total of 7767 studies were included from the database searches, and 104 studies from handsearching and citation searching. After all duplicates were removed, a total of 5919 studies were imported into Covidence to undergo title and abstract screening. A total of 74 studies were included in the scoping review. The search outcomes can be found in the PRISMA flow chart in figure 1.

### Quantitative and qualitative study outcomes

#### General study characteristics

The included studies (n=74), based on the selection criteria, varied in study designs. A descriptive summary for each of the included studies can be found in online supplemental 3. Of the included studies, 31 (41.89%) were qualitative studies, 24 (32.43%) were quantitative studies and 19 (25.68%) were mixed-methods studies. In terms of the year of publication, 59 (79.73%) studies were published between 2010 and 2024, while only 15 (20.27%) studies were published during the years 2000 to 2009. The largest number of studies came from the AMRO/PAHO region, with 21 (28.38%) studies. The AFRO region had the second highest frequency with 19 (25.68%) published studies, and the least number of studies were in the EMRO and SEARO regions, with only 2 (2.70%) and 3 studies (4.05%), respectively. While the frequency of published CSE educator training studies varied across the regions, there was an almost equal balance between LMICs and HICs, with 36 (48.65%) and 38 (51.35%) studies, respectively.

The target population of the included studies varied between preservice teachers, in-service school teachers, school counsellors and school nurses. Only 9 (12.16%) studies targeted preservice teachers, while 54 (72.97%) studies focused on in-service teachers. Only 1 (1.35%) study focused exclusively on school counsellors, and none of the studies targeted school nurses alone. However, although school counsellors and nurses were rarely the sole focus, 10 (13.51%) studies reported their target population as more than one population, meaning school counsellors and nurses were trained with teachers.

Training methods also varied across studies. In the 9 (12.16%) studies that focused on preservice teachers, training was done through university courses and used techniques such as simulation games and practice teaching sessions. Most of the studies, 46 (62.16%), reported in-person training methods. Different approaches were used when conducting in-person training methods, which included the use of training manuals, team-based learning (TBL) techniques, networking activities, practice teaching sessions and informational videos. A few studies, 9 (12.16%), reported using more than one training method, which means they used a combination of in-person and online. When used in combination with in-person training methods, online methods were used to train and offer supplemental support to educators before and after classroom implementation through teaching manuals, informational videos, a mobile app and e-coaching. Additionally, one study reported letting educators train at their own pace before meeting for in-person training. All study characteristics are detailed in table 1.

**Table 1:** Study characteristics (n=74)

Characteristic	Category	n	%
Study design	Qualitative study	31	41.89
	Quantitative study	24	32.43
	Mixed-methods study	19	25.68
Publication year	2000–2004	5	6.76
	2005–2009	10	13.51
	2010–2014	18	24.32
	2019	22	29.73
	2020–2024	19	25.68
WHO region	AFRO	19	25.68
	AMRO/PAHO	21	28.38
	EMRO	2	2.70
	EURO	15	20.27
	SEARO	3	4.05
	WPRO	14	18.92
Country income status	LMIC	36	48.65
	HIC	38	51.35
Population	Preservice teachers	9	12.16
	In-service teachers	54	72.97
	School counsellors	1	1.35
	School nurses	0	0.00
	More than one	10	13.51
Training method	Preservice university course	9	12.16
	In-person	46	62.16
	Online	1	1.35
	More than one	9	12.16
	Unspecified	9	12.16

AFRO, African; AMRO/PAHO, Americas; EMRO, Eastern Mediterranean; EURO, European; HIC, high-income country; LMIC, low- and middle-income countries; SEARO, Southeast Asia; WPRO, Western Europe and Central Asia.

#### Training durations

The duration of the examined training programmes varied widely, ranging from 1 day or less to semester-based courses and programmes extending over months or years. To improve consistency, training durations were categorised according to how they were reported in the original studies, and each study was assigned to one mutually exclusive duration category. Studies reporting day-based durations were grouped as short duration (1 day or less), multiday duration (2–5 days) or medium duration (6–14 days). Programmes reported as extending over months to years were classified as long duration. Preservice university-based courses were classified separately because they represented a distinct training format rather than a discrete in-service training period. Studies reporting only contact hours were retained in a separate hours-based category. Studies with mixed, variable, or otherwise unclear duration formats were classified as ‘other’, while studies that did not specify duration were categorised as ‘unspecified’. Multiday duration was the most frequently reported category, accounting for 19 studies (25.68%). University-based courses accounted for 9 studies (12.16%), most of which were semester-based. A separate hours-based category included 3 studies (4.05%) reporting durations of 50–100 contact hours. Four studies (5.41%) were categorised as ‘other’ because they described duration in ways that were not directly comparable, such as continued education programmes, professional development sessions, ‘several days’, or different training durations for different participant groups. Full training duration details are presented in table 2.

**Table 2:** Training durations as described in the included studies (n=74)

Category	Training durations	n	%
Short duration	1 day or less	9	12.16
Multiday duration	2–5 days	19	25.68
Medium duration	6–14 days	7	9.46
Long duration	Months to years	5	6.76
University courses	Semester-based or course-based training	9	12.16
Extended hours	50–100 contact hours	3	4.05
Other	Differently reported durations	4	5.41
Unspecified durations	Unspecified	18	24.32

#### Reoccurring trainings

Several studies reported a total of nine recurring training programmes, which included Sexual Health and Relationships: safe, happy, and responsible, Long Live Love (LLL) or (LLL+), Family Life and HIV Education, Health and Family Life Education, Reproductive Health Education/ Sexuality Education Program (RHE/SE), Sexuality Education Academy, ‘Health Day’, Sex Abuse Treatment Center Training Program and the Big Decisions programmes. These trainings targeted different populations and covered different CSE topics. The majority of these recurring training programmes occurred in the same region; seven studies and two studies reported implementing training across two separate regions. Either the training programmes that took place in different regions took place in a culturally similar region, or the training programme was adapted to fit the different culture in which it was being implemented. A detailed list of all the recurring programmes can also be found in table 3.

**Table 3:** Recurring trainings reported throughout the included studies

Training name	Occurrences	Region
Sexual Health and Relationships: safe, happy and responsible	4	EURO
Long Live Love (LLL) or (LLL+)	3	EURO
Family Life and HIV Education	2	AFRO
Health and Family Life Education	2	AMRO/PAHO
Reproductive Health Education/ Sexuality Education Program	2	AFRO and SEARO
Sexuality Education Academy	2	AMRO/PAHO
Health Day	2	EURO
Sex Abuse Treatment Center Training Program	2	AMRO/PAHO
Big Decisions	2	AFRO and AMRO/PAHO

AFRO, African; AMRO/PAHO, Americas; EURO, European; SEARO, Southeast Asia.

#### Quantitative study outcomes

43 studies (24 quantitative and 19 mixed methods) were included in the quantitative analysis. Several patterns were identified from the quantitative studies, which included CSE topics covered in training programmes, outcomes of training programmes, implementation fidelity, and training evaluation.

#### CSE topics covered in the training programmes

Throughout the training programmes, educators were trained on various topics related to CSE. Related topics were categorised into groups, resulting in eight CSE topics, as presented in table 4. The primary CSE topics, encompassing SRH rights and education and risky sexual behaviour (contraception methods, pregnancy, abortion and family planning), were most frequently reported, with 15 (34.88%) studies addressing both topics. HIV/AIDS and STIs were addressed in 12 (27.91%) training programmes, and decision-making and safety were covered in 10 (27.91%) of the examined training programmes. Relationships topic was reported in nine (20.93%) training programmes. Sexual violence/abuse, victim blaming attitude and substance abuse were mentioned in eight (18.60%) studies, and gender equality and diversity were addressed in six (13.95%) different studies. Only three (6.98%) training programmes focused on some aspect of abstinence.

**Table 4:** CSE topics covered in the training programmes of quantitative and mixed methods studies (n=43)

CSE topics covered in training programmes	n	%*
Sexual and reproductive health rights and education	15	34.88
Risky sexual behaviour (contraception methods, pregnancy, abortion and family planning)	15	34.88
HIV/AIDS and STIs	12	27.91
Decision making and safety	10	23.26
Relationships	9	20.93
Sexual violence/abuse, victim blaming attitudes and substance abuse	8	18.60
Gender equality and diversity	6	13.95
Abstinence	3	6.98

* The total number of percentages (%) will not equal 100 because not all quantitative and mixed-methods studies covered all the mentioned CSE topics, or some studies covered more than one. This number just represents the percentage of studies that did cover a certain topic(s) out of the 43 quantitative and mixed-methods studies.

CSE, comprehensive sexuality education; STIs, sexually transmitted infections.

#### Outcomes of training programmes

Educator outcomes of the different CSE training approaches and CSE topics were assessed through four different methods (table 5). 16 studies (37.21%) assessed educator confidence and comfort levels. Furthermore, 16 (37.21%) studies assessed knowledge gains. In 12 (27.91%) studies, educators reported benefitting from the training by learning new facilitation skills, student activities and different approaches to teaching CSE. Shifts in educator attitudes were assessed in eight (18.60%) studies.

**Table 5:** Outcomes of CSE training programmes in quantitative and mixed-methods studies (n=43)

Outcomes of CSE training programmes	n	%*
Confidence/comfort increase	16	37.21
Knowledge gains	16	37.21
Reported benefits from the training: facilitation skills, activities, approaches to teaching CSE	12	27.91
Shifts in attitudes	8	18.60

* The total number of percentages will not equal 100 because some studies assessed more than one educator outcome. This number just represents the percentage of studies that assessed certain educator outcomes out of the 43 quantitative and mixed-methods studies.

CSE, comprehensive sexuality education.

#### Training and implementation fidelity

Training fidelity refers to whether the training was modified by trained educators prior to classroom implementation to better align with school settings.^44^ Two studies (4.65%) reported that training fidelity occurred because of gatekeepers such as religious leaders who influenced policymakers to alter the training. Implementation fidelity refers to the degree to which the CSE intervention or curriculum was implemented in the classroom after educators received training.^44^ Study outcomes revealed five different factors that influenced implementation fidelity (table 6). Time limitations were reported as the biggest barrier to implementation fidelity, as indicated in eight (18.60%) studies. Support from school/administrators was reported in six studies (13.95%), and parental support was reported as an influencing factor on implementation fidelity in four (9.30%) studies. Some CSE lessons were altered because educators did not think the curriculum was appropriate or did not align with personal beliefs, and this was reported in four (9.30%) studies. Only two (4.65%) studies reported a lack of resources as an influence on implementation fidelity, and this was reported as either a lack of empty classrooms or no available computers.

**Table 6:** Training and implementation fidelity in quantitative and mixed-methods studies (n=43)

Training and implementation fidelity	n	%*
Time limitations	8	18.60
School/administration support	6	13.95
Parental support	4	9.30
Inappropriate curricula (role play)	4	9.30
Lack of resources	2	4.65
Gatekeepers	2	4.65

* The total number of percentages (percentage) will not equal 100 because not all studies acquired training and implementation fidelity. This number just represents the % of studies that did report training and implementation fidelity out of the 43 quantitative and mixed-methods studies.

#### Supplemental training methods

Some studies had educators evaluate supplemental training methods. Technical support strategies, which included follow-up training and feedback support from the training staff and school administrators, were reported as positive supplemental training methods in five (11.63%) studies. Evaluation of training duration was measured in two (4.65%) studies. Lastly, two (4.65%) studies had educators take part in networking activities during and after training and then asked them to evaluate this part of the training. Positive feedback was observed towards networking as it provided educators with new partnerships.

### Qualitative study outcomes

50 studies (31 qualitative and 19 mixed methods) were included in the qualitative analysis. Four main themes were identified from these studies. The themes were as follows: barriers to CSE training, impacts of CSE training on educators, facilitators of CSE implementation, and barriers to CSE implementation. Each of these four themes included several subthemes, as shown in table 7.

**Table 7:** Qualitative themes and subthemes

Theme	Subtheme
Barriers to CSE training	Lack of preservice training
	Inadequate training
	Lack of institutional support
Impacts of CSE training on educators	Pretraining expectations and needs
	General feedback
	Knowledge gains
	Increased confidence
	Self-awareness and attitude shifts
Facilitators of CSE implementation	Instructional strategies
	Parental support
	Institutional support
	Networking opportunities
Barriers to CSE implementation	Resistance from educators
	Lack of parental support
	Lack of institutional support
	General implementation challenges

CSE, comprehensive sexuality education.

#### Barriers to CSE training

While CSE is important for adolescent health, there were still barriers to effectively training educators to deliver CSE curricula. These barriers were a lack of preservice training, inadequate training, and lack of institutional support.

#### Lack of preservice training

Lack of preservice training was mentioned in several different studies as a reason why several in-service teachers did not implement CSE in the classroom. Some teachers stated that they did not have preservice training at all, while others who had received this training did not feel it was adequate in preparing them to implement a CSE curriculum.

Teachers admitted they received little preparation on the subject of SE when they studied their professional careers.^45^

#### Inadequate training

Another barrier identified was the inadequate training programmes provided to educators. In some studies, teachers felt that even though they had been offered some form of CSE training, it still did not meet their needs regarding how to implement CSE in the classroom. This was either because they were not taught the proper skills to disseminate this knowledge or because they had not received sufficient resources to assist in CSE implementation. Another study that examined how teachers were trained on CSE across different countries found that although teachers were being trained, they were not being taught all SE topics.

#### institutional support

A final barrier was identified as the lack of institutional support. Different studies found that the Ministry of Education (MoE) played a role in whether educators could partake in CSE training, and some studies found that even though the MoE had sexual reproductive education (SRE) textbooks available, they did not offer any supplemental educator training to learn how to use these textbooks. Some studies found institutional support heavily relied on the availability of financial resources, and if authorities found the training to be of importance.

##### Impacts of the CSE training on educators

This theme identified the studies where educators had been trained on certain CSE programmes and evaluated their outcomes. The subthemes that emerged from this theme were pretraining expectations and needs, knowledge gains, increased confidence, self-awareness and attitude shifts.

### Pretraining expectations and needs

Before starting certain training programmes, some studies asked participants to express what they had hoped to receive from the training. The pretraining expectations and needs varied among educators, with some wanting to just extend their SE knowledge and others wanting specific outcomes from the proposed training.

Teachers expressed hopes of increasing their knowledge of sexuality and HIV, and gaining dynamic approaches to effectively and confidently teach sex education to Grade 8 students.^46^

### General feedback

After receiving training, some studies asked educators to provide general feedback on the overall training programme they participated in. Most educators provided positive feedback and some even highlighted certain aspects of the training programme that they appreciated, such as cultural sensitivity and acquiring new teaching skills.

One Participant expressed an appreciation for using Ecuadorian-specific data and resources as seen in the comment: “[I enjoyed Day 3’s theme] because it was very focused on the country’s problems, tools for young people for the prevention of unwanted pregnancy and STIs.” Still others reported enjoying having learned something new, though culturally sensitive, as expressed in the comment: “Gender and sexual diversity, since it’s something culturally new.^47^

### Knowledge gains

Studies also evaluated knowledge gains after certain training programmes. Post-training knowledge gains were often measured through quantitative methods; however, some studies highlighted certain aspects of knowledge gain through post-training interviews. Some of these knowledge gains occurred because of certain training methods that were used during educator training, such as games, homework and infographic videos. In addition, educators stated they even acquired new skills in how to teach certain CSE topics. Some educators left the training with new abilities.

### Increased confidence

Some studies showed that after receiving CSE training, there was an increase in confidence among educators in their abilities to implement CSE in the classroom. This increase in confidence came from educators learning how to access new CSE resources. In some studies, the increase in confidence also helped educators overcome personal discomfort in discussing CSE in front of students.

### Self-awareness and attitude shifts

Some studies described the impact of training on educators as bringing about self-awareness and attitude shifts. A few studies described training as helping to modify preconceived ideas they held about CSE and certain CSE topics like contraceptive methods. Additionally, training helped educators realise the significance of CSE in schools and their role as SE educators. It also showed how training added to educators’ personal growth.

### Facilitators of CSE implementation

Studies that evaluated CSE implementation after training was given to educators found instructional strategies, parental support, institutional support and networking with other educators to be facilitating factors to CSE implementation.

### Instructional strategies

The biggest facilitating factor of CSE being implemented in the classroom was found in several studies to be whether educators had gained adequate instructional strategies from the training they received. These instructional strategies determined how and to what extent educators were teaching CSE, for example, using manuals or learning new activities to incorporate into the SE curriculum.

One of the cultural barriers that educators overcame was to use the new approaches to teach about masturbation.^48^

### Parental support

Parental support was also recognised as a facilitating factor. Some educators felt that clear communication with parents about any SE content was necessary when trying to implement a successful CSE curriculum because it allows them to support these ideas at home.

Teachers felt clear communication with parents was an effective method of addressing parental concerns and enhancing the success of an RSE program: I think the responsibility about educating the parent would be a great program to have. I think parents at this school are eager to know how they can help.^49^

### Institutional support

Institutional support, especially from principals and school management, was highlighted as an important facilitating factor in the implementation of CSE programmes. This support causes a sense of ownership and motivation among educators to overcome challenges in CSE implementation. Several studies showed the role of leadership in facilitating CSE training and implementation.

Some teachers mentioned that the sessions can be sustained in individual schools if the head teachers actively support teachers by regularly providing them extra class time and schedule in the routine as they did during the intervention period.^50^

### Networking opportunities

In a few studies, collaboration and networking among educators were key factors in strengthening the delivery of CSE curricula. Educators relied on others for support, sharing resources and strategies to enhance CSE delivery. Networking opportunities not only helped increase educators’ knowledge but also gave them a sense of ownership and teamwork.

### Barriers to CSE implementation

Several barriers to CSE implementation were identified when educators were examined after participating in CSE training. The subthemes that emerged were resistance from educators, lack of parental support, lack of institutional support and general implementation challenges.

### Resistance from educators

The main barrier to implementation was resistance from educators. This resistance stemmed from different reasons, such as certain instructional strategies not being seen as appropriate for the classroom, like role play. Additionally, in some cases, educators still addressed CSE topics based on their personal beliefs and did not relay the message they were taught in training.

When asked which values they were likely to transfer when teaching the curriculum, the majority stated that it is the belief that abstinence is best and that no sex before marriage should be upheld.^46^

### Lack of parental support

While parental support has been recognised as a facilitating factor in some studies, the lack of parental support has also been recognised as a barrier to CSE implementation in others. In some studies, educators recognised that parents do not always agree with CSE curricula being taught to their children. They have expressed their worries about these objections potentially causing negative backlash.

### Lack of institutional support

Previously, institutional support was found to be a facilitating factor; however, some studies have recognised a lack of institutional support as a barrier to CSE implementation. Educators have recognised it as a barrier mainly because it is not seen as an important topic; therefore, it is not given priority like other subjects.

Health education teachers indicated that not having a dedicated or permanent classroom space assigned to teach health education not only impacts their ability to deliver the sexual health lessons, but it also heightens student discomfort with the lesson content.^51^

### General implementation challenges

In addition to the aforementioned factors, which act as barriers to implementation, educators have mentioned several other factors as general implementation challenges. The main challenge to implementing CSE in the classroom was time. Several studies identified this in postimplementation interviews with educators. Other factors that contributed to general implementation challenges included the lack of classroom resources, technological issues, the fact that the CSE content did not match the school’s science curricula and the way the classrooms were set up.

## Discussion

The results of this scoping review highlight the different training methods for CSE. These methods varied widely in their approaches and effectiveness, with preservice university courses demonstrating the strongest impact on educators’ professional development and preparedness. In-person trainings that incorporate manuals, TBL, practice teaching and informational videos consistently improved confidence and implementation of fidelity. However, exclusively online methods showed limitations, particularly in promoting essential social skills. Combination training that blended online and in-person components offered promising benefits but exhibited mixed results when e-coaching was used. Regional disparities resulted, with research concentrated in the AMRO/PAHO, EURO and WPRO regions. Significant gaps in EMRO and SEARO settings emerged, which were often a result of cultural and religious sensitivities. The recurrent use of adaptable training programmes like LLL/LLL+and Big Decisions highlighted the importance of cultural tailoring. While training commonly covered WHO-recommended and UNESCO-recommended CSE topics, barriers such as inadequate preparation, institutional resistance, time constraints and educator discomfort persisted. In contrast, facilitators, including instructional strategies, technical support, networking opportunities and stakeholder engagement, proved detrimental in enhancing implementation fidelity and educator confidence.

### Training methods and effectiveness

#### Preservice university courses

Although only a small number of studies reported training preservice university teachers, this method has been proven to show the greatest influence on their professional development, improving both skills and creative abilities by instilling values, knowledge and attitudes required to deliver quality education.^19 52^ The effects of preservice training were evident after a 1-day ‘Health Day’ training, where distinguished attitude shifts occurred among participants. Moreover, training methods such as simulation games and practice teaching sessions were found to significantly increase preservice teachers’ confidence and satisfaction towards CSE training and implementation. These outcomes align with previous research that has identified confidence and satisfaction as influencing factors of training success and preparedness for effective curricula implementation.^17 53^ By acquiring these qualities early in their careers, preservice training provides a solid foundation for effective CSE implementation.

#### In-person

Different techniques were described when reporting in-person training methods. Successful techniques included the use of training manuals, TBL strategies, networking opportunities, practice teaching sessions and informational videos. Training manuals increased educators’ confidence levels by providing them with a structured guide that outlined the training programme’s aims and objectives and CSE content while also preparing them with lesson plans, discussion topics and different teaching techniques to increase implementation fidelity. Manuals have been found to be effective preparation methods because they can be delivered in different forms and can be easily updated, making information easily accessible at any time.^54^ Furthermore, preparedness levels have been found to be associated with boosts in confidence levels and increases in implementation fidelity.^21^ Manuals enable educators by preparing them to provide consistent and accurate information, thus enhancing implementation fidelity.

TBL approaches that included the combined training of school teachers and nurses resulted in significant knowledge gains and self-efficacy increases. Previous studies have supported the use of TBL approaches when implementing knowledge-based training because when group members can relate to one another through shared experiences, the effectiveness of training increases.^21 55^ Similar to TBL approaches, networking opportunities provided educators with extra human resources, such as other educators, post-training to assist in lesson planning and offer feedback. Networking allows educators to try out new ideas and motivates them to grow professionally.^56^ More recent studies have found that both TBL approaches and networking activities improve collegiality among educators while addressing a shared pedagogical focus.^57 58^ Implementing TBL approaches during training and providing networking opportunities post-training allows for effective training to be carried out and improved implementation fidelity.

Practice teaching sessions were found to help educators prepare for lessons in advance by allowing them to rehearse lesson plans, specifically ones that addressed sensitive topics, and prepare for any questions that students may have. Practice-based training has been found to be effective when implementing new curricula because it allows educators to prepare new content, which also boosts confidence levels.^21 59^ Providing educators with practice teaching sessions during training enhances educator preparedness levels, which in turn increases confidence levels and implementation fidelity.

Informational videos that offered factual information through animations and shared experiences were provided in a large number of reported trainings and implementations. Not only has previous research found informational videos to be an effective training method for educators’ professional development, but it has also found that many educators prefer this method when training and when implementing curricula in the classroom.^59 60^ The use of informational videos provides accurate information on sensitive topics that some educators might have difficulty speaking about, or students might find awkward, and cause disruptive behaviour.^61^

#### Online

Only one study reported using an exclusively online method for training and implementation. This training method was a reformed version of the LLL/LLL+programme. Although outcomes met the programme’s goals and overcame potential barriers, the findings indicated that it lacked the acquisition of social skills. The acquisition of social skills has been shown to be a key factor in effectively teaching and learning about CSE and creating supportive and caring classroom environments.^62^ The majority of CSE training and curricula are developed on the basis of social cognitive theory, which means people learn by observing others and should include interactive methods to promote cognitive learning and critical thinking.^63^ Moreover, potential issues with virtual learning have been shown to include the lack of social interaction.^64^ An exclusively online CSE training and implementation takes away from the social interaction components that make up an effective CSE training and implementation.

#### Combination training: online and in-person

Combination training methods included both in-person and online forums, which were used interchangeably at different stages, sometimes pretraining, sometimes during, and sometimes post-training. Material preparation is one of the easiest ways to save time and reach ultimate efficiency in learning environments, pretraining.^65^ Pretraining reflections via online forums have been recommended to help educators promote interaction and engagement before attending in-person training.^66^ Pretraining online methods could enhance training outcomes by ensuring educators are equipped with basic CSE knowledge and preparing them for interaction and engagement with discussion topics.

Educator motivation is enhanced when they are provided with the opportunity to participate in open dialogue during training.^58^ Networking opportunities provided via social media platforms give educators the chance to connect with other participating educators during CSE training. Follow-up training sessions and access to online resources and curricula via online forums post-training have been found to assist educators in preparing for effective implementation. Furthermore, this increases educators’ confidence levels and enhances implementation fidelity.^21^ Unfortunately, the implementation of an e-coach post-training was not found to have a significant effect on implementation fidelity. The failure of a supplemental e-coach method might be a result of educators not using it or using it insufficiently, which can cause the failure of an experimental implementation method.^67 68^

### Training durations

Training durations reported in this study’s findings spanned from less than a day to years, with the addition of university courses being presented as a training duration category. The reason for mixed recommendations of training duration in the findings could be because studies suggest that the duration of a training should reflect on the training’s context as well as its objectives.^69^ Therefore, one set of recommendations cannot be provided for all training. Nonetheless, research shows support for training programmes that are spread over a semester and usually include some form of follow-up education.^70^ This data provides further evidence that supports that the best time to train educators is during preservice training, as it provides them with the most effective skills and creative abilities. Additionally, researchers You, Park, Hong and Warren suggest that the most effective duration for science professional development for in-service educators is less than 48 hours and should be held over a period of under 3 months.^20^

### Regional variations

Findings revealed a gap in CSE training research across regions, with the majority of studies being conducted in the AMRO/PAHO, EURO and WPRO regions and the least number of studies in the EMRO and SEARO regions. The underrepresentation of CSE studies in the EMRO and SEARO regions could be the result of sensitive cultural and religious backgrounds. The majority of the countries that make up these regions follow conservative Islamic beliefs.^71^ Sexual health topics in these parts of the world are usually viewed as taboo subjects and are rarely discussed, specifically in an educational setting with unmarried students.^72^,^75^ Fair representation of the AMRO/PAHO, EURO and WPRO regions could be attributed to strong institutional frameworks and supportive policy frameworks.^76^

### Reoccurring trainings

Nine different training programmes reoccurred across several different studies. The majority of the trainings that reoccurred throughout multiple studies were conducted in the same region and sometimes slightly modified. The LLL/LLL+programme was repeated three different times; still, each time used a different training and implementation method. Additionally, the versions that included some form of online training or implementation did not succeed as well as the all-in-person training did. This may be because important social skills were not acquired, or the use of an e-coach was not implemented sufficiently.^62 67 68^ The Big Decisions training programme reappeared across two studies in different regions. The reason for this is most likely that the Big Decisions programme could be modified to include cultural sensitivity. This key feature is a key indicator of a successful CSE programme, specifically in culturally and religiously sensitive environments.^77^ Additionally, the RHE/SE programme reoccurred twice across two different regions, India and Nigeria. Despite the different geographical and cultural backgrounds, researchers chose to implement this training programme in these countries because they both suffered from the same reproductive health issues. The results of this study provide valuable insight for implementing a scalable training programme in settings with different backgrounds to address the same issues.

### CSE topics and outcomes

The different CSE topics that were covered during educator trainings included SRH rights and education, risky sexual behaviour (contraception methods, pregnancy, abortion and family planning), HIV/AIDS and STIs, decision-making and safety, relationships, sexual violence/abuse, victim-blaming attitudes and substance abuse, gender equality and diversity, and abstinence. These topics were used to implement knowledge, alter confidence levels and shift the attitudes of educators. These topics align with the WHO and UNESCO recommendations for CSE curriculum topics.^3 78^ Proper implementation of these topics during training prepares and encourages educators to talk about topics they previously had little knowledge about or did not feel comfortable talking about to students. Subsequently, this results in higher degrees of implementation fidelity.

### Barriers to CSE training

Findings indicated key influencing factors affecting barriers to training included the lack of preservice training, inadequate training and institutional resistance. Several educators attributed the lack of preservice training to feelings of discomfort and resistance to teaching CSE curricula. These findings align with previous studies that reported CSE trainings were limited for preservice educators and when they were offered, students were not given the opportunity to apply them in a practical teaching setting.^79 80^ Inadequate training due to not being trained on all CSE-related topics and limited resources was also identified as a barrier to training. These results align with previous research outcomes that suggest the use of multitraining methods when developing an effective training programme for educators.^20 21 81^ However, after evaluating the different programmes in this review, it was clear that not all programmes used the suggested multi-methods, further highlighting the need to address these gaps and modify or develop new training. Research has also indicated that due to the wide range of CSE topics with the diverse targeted communities, developing a set programme would be impossible.^69 82^ This data further supports the need for adaptable training programmes to tailor to the needs of educators in culturally sensitive settings. The lack of institutional support due to government involvement and resource constraints was also found to be a barrier identified by educators. These findings match previous study outcomes that have reported institutional restrictions are influenced by policies and access to resources, thereby influencing access to training.^83^,^85^

### Facilitators and barriers to CSE curricula implementation

Facilitating factors identified in this review included newly acquired instructional strategies, like the use of manuals and drawing-led sessions, as well as technical support strategies, such as feedback, follow-up support and networking opportunities. Several studies have reported the claim that effective instructional strategies increase classroom implementation, subsequently leading to increased levels of preparedness and comfort in educators and better student learning outcomes.^2154 86^,^88^ Feedback and follow-up support post-training increased implementation fidelity significantly by allowing educators to ask questions and adjust the curricula. Networking opportunities were also found to assist educators by providing feedback on lesson plans and teaching techniques. These findings align with study outcomes that have indicated technical support strategies and networking opportunities to be made to increase curricula implementation.^20 21 81 89^ Barriers to CSE curricula implementation were identified as resistance from educators due to impractical instructional strategies, CSE curricula that did not align with personal beliefs, and mixed-gender classrooms. Similar findings have been reported in several studies, which indicated that educators cause bias when implementing certain CSE curricula because of religious beliefs, implement changes because of discomfort towards instructional strategies, and the structure of mixed-gendered classrooms.^90^,^94^ Another major barrier that was reported in studies was time constraints because many educators did not feel they had enough time to include all the CSE curricula. Additionally, in many countries, CSE is not an examinable subject; therefore, educators do not prioritise time to implement CSE curricula.^95^ This finding further underscores the importance of developing national guidelines and mandates. Parental and institutional support were identified as both facilitating factors and barriers to CSE implementation. Previous studies have highlighted the significant contributions parents and school administrators make to CSE programmes and their influence on implementation.^91 94 96^ Studies have found, CSE curricula that incorporate parent participation have been proven to increase adolescent intentions to postpone sexual intercourse and use contraceptives.^97 98^ These findings make it evident that to encourage CSE training and full implementation, stakeholders such as institution administrators and parents need to cooperate in CSE implementation and provide effective feedback.

### Recommendations

Based on the findings of this review, practical and policy recommendations for the development and implementation of efficient CSE educator training programmes have been identified to address common barriers.

The following recommendations have been made to enhance the delivery of CSE educator training:

1. To expand the development of preservice training programmes across all degree programmes.
2. To develop structured manuals that will assist educators in facilitating CSE implementation.
3. To develop a combination training of in-person and online sessions.
4. To develop CSE training programmes that incorporate the use of group learning and provide networking opportunities for educators during and after training.
5. To implement CSE curricula in gender-separate classrooms.
6. To ensure adequate training durations are established.
7. To develop CSE training programmes that allow for adaptation across various regions that contain diverse cultural, religious, socioeconomic and geographical backgrounds while still including all CSE topics and maintaining core values.

By implementing these changes, educators’ knowledge, confidence, and pedagogy skills could potentially increase student learning outcomes would also increase.^17 52 54^

The following policy recommendations have been made to eliminate research gaps and potentially improve CSE educator training outcomes:

1. CSE needs to be a mandated core curriculum for all preservice educators across all degree programmes.
2. Culturally sensitive regional guidelines should be developed to address diverse cultures, religions and social norms.
3. Governments should prioritise funding for CSE training programmes and implementation.
4. Governments need to mandate the implementation of CSE curricula in schools.
5. Governments need to monitor and evaluate all CSE training and implementation.

### Strengths and limitations

This scoping review demonstrated many strengths. The review was guided by a protocol approved by senior academic researchers, a structured conceptual framework, and the Arksey and O'Malley mixed methods scoping review design.^35^ To ensure all relevant literature was included, a comprehensive search strategy was implemented throughout six different online databases, three hand searches, and one citation search. Only peer-reviewed studies were included to ensure reliability and validity. Date limitations helped narrow the focus of the review by ensuring current implementation challenges, up-to-date educational approaches, and advancements in technology were included. By applying the Arksey and O’Malley mixed-methods design, this review was able to provide a holistic view of the relevant research. Quantitative studies provided statistical analyses of numerical data and were further supported by qualitative findings through individual perspectives. Lastly, by including global studies, researchers were able to assess worldwide barriers and facilitators to CSE training and classroom implementation. This assessment provided clear directions for adaptation methods for future training and classroom implementation. While the results of this review provide valuable insights into CSE educator training, there were a few limitations. This review may be subject to selection bias and publication bias because of language restrictions and the exclusion of grey literature. Language restrictions applied to the study selection process could have potentially excluded relevant research published in languages other than Arabic and English. The exclusion of grey literature, such as reports, policy documents, or conference papers, could have overlooked valuable insights provided by local practices, emerging trends or unpublished studies.

## Conclusions

This scoping review mapped the current state of knowledge and gaps in CSE educator training, including training methods, content, barriers to training, and facilitators and barriers to implementation. Key findings of this review included effective training methods and durations, in addition to highlighting the importance of CSE topics and the impact they have on educator knowledge, confidence levels, and attitudes. The findings also revealed the necessity to address research gaps in underrepresented regions, such as EMRO and SEARO, and propose practical and policy recommendations to enhance CSE educator training. Addressing these barriers will allow educators to receive adequate training in CSE, further enhancing overall CSE education and SRH services for students.

## Supplementary material

10.1136/bmjopen-2025-111342 online supplemental file 1

10.1136/bmjopen-2025-111342 online supplemental file 2

10.1136/bmjopen-2025-111342 online supplemental file 3

## Data Availability

All data relevant to the study are included in the article or uploaded as supplementary information.
