# Supplementary material for: Addressing the gaps: a mixed-methods scoping review on comprehensive sexuality education training for school educators
Source: BMJ Open. 2026 Jun 19;16(6):e111342. doi: 10.1136/bmjopen-2025-111342 (PMC13289112; doi:10.1136/bmjopen-2025-111342)
Supplement: online supplemental file 2 [file bmjopen-16-6-s002.docx]

# Supplement 2: Search Strategy for each included database

| **Search Terms** | **Database** | **Limitations/Filters** | **Date** |
| --- | --- | --- | --- |
| ((("Comprehensive Sexual Education" ) OR ("Sexual Education" ) OR ("Sexual reproductive health*" )) AND ((Training ) OR (Learning ) OR ("Professional* Development" ) OR (Education ) OR (Workshop )) AND ((Teacher* ) OR (Educator ) OR (Counselor ) OR (Nurse* ) OR ("Field* officer" )) AND ((School ) OR ("School based" ))) | PubMed [NLM] | None | April, 2024 |
| ((("Comprehensive Sexual Education" ) OR ("Sexual Education" ) OR ("Sexual reproductive health*" )) AND ((Training ) OR (Learning ) OR ("Professional* Development" ) OR (Education ) OR (Workshop )) AND ((Teacher* ) OR (Educator ) OR (Counselor ) OR (Nurse* ) OR ("Field* officer" )) AND ((School ) OR ("School based" ))) | WebofScience [Clarivate] | None | April, 2024 |
| ((("Comprehensive Sexual Education" ) OR ("Sexual Education" ) OR ("Sexual reproductive health*" )) AND ((Training ) OR (Learning ) OR ("Professional* Development" ) OR (Education ) OR (Workshop )) AND ((Teacher* ) OR (Educator ) OR (Counselor ) OR (Nurse* ) OR ("Field* officer" )) AND ((School ) OR ("School based" ))) | Scopus [Elsevier] | None | April, 2024 |
| ((("Comprehensive Sexual Education" ) OR ("Sexual Education" ) OR ("Sexual reproductive health*" )) AND ((Training ) OR (Learning ) OR ("Professional* Development" ) OR (Education ) OR (Workshop )) AND ((Teacher* ) OR (Educator ) OR (Counselor ) OR (Nurse* ) OR ("Field* officer" )) AND ((School ) OR ("School based" ))) | Embase [Ovid] | None | April, 2024 |
| ((("Comprehensive Sexual Education" ) OR ("Sexual Education" ) OR ("Sexual reproductive health*" )) AND ((Training ) OR (Learning ) OR ("Professional* Development" ) OR (Education ) OR (Workshop )) AND ((Teacher* ) OR (Educator ) OR (Counselor ) OR (Nurse* ) OR ("Field* officer" )) AND ((School ) OR ("School based" ))) | CINAHL/ APA PsychInfo/ APA PsychArticles [EBSCOhost] | None | April, 2024 |
