# Supplementary material for: Addressing the gaps: a mixed-methods scoping review on comprehensive sexuality education training for school educators
Source: BMJ Open. 2026 Jun 19;16(6):e111342. doi: 10.1136/bmjopen-2025-111342 (PMC13289112; doi:10.1136/bmjopen-2025-111342)
Supplement: online supplemental file 3 [file bmjopen-16-6-s003.docx]

Supplement 3: Descriptive Summaries of the Included Studies

| Citation | Country | Study Design | Target Population | Aim of study | Study Results |
| --- | --- | --- | --- | --- | --- |
| Acharya et al., 2014 (84) | India | Mixed methods study | teachers | To assess the effect of training program on teachers' knowledge of and attitude towards reproductive health education /sexuality education [RHE/SE] in five randomly selected schools in rural area of Wardha district, Maharashtra, India. | A significant increase in percentage of those who had good knowledge in general areas of RHE/SE at post-training assessment & pre-post attitudinal disposition assessments show that there was an increase in percentage of those who were favorably disposed to the teaching of RHE/SE. |
| Adegbenro et al., 2006 (85) | Nigeria | Quantitative study | teachers | To assess the effect of training program on teachers' knowledge of and attitude towards reproductive health education /sexuality education [RHE/SE] in five randomly selected rural schools in Ife-North local government area, Southwest, Nigeria. | Results showed a significant increase in percentage of those who had good knowledge in general areas of RHE/SE and attitudinal disposition assessments show that there was an increase in percentage of those who were favorably disposed to the teaching of RHE/SE in Nigeria Schools. |
| Ahmed et al., 2006 (86) | South Africa | Qualitative study | teachers | To describe the results of a qualitative process evaluation of a teacher training program which forms part of a larger HIV/acquired immunodeficiency syndrome (AIDS)intervention project. It provides a description of the knowledge, skills and confidence with which teachers equipped themselves during the training. | Findings indicate that teachers reported increased confidence and comfort in teaching the sexuality curriculum. However, many struggled with the transfer of sexual reproductive knowledge and facilitative teaching methods into the classroom context. |
| Alavi-Arjas et al., 2018 (87) | Iran | Quantitative study | school counselors | To assess the effectiveness of educational intervention on girls’ high school counselors' knowledge on, and self-efficacy of, adolescent SRH. In addition to this main goal, this study compared the effect of the two different learning approaches: lecturing and TBL | The final mean of counselors' knowledge and self-efficacy scores in the team-based learning (TBL) group were significantly higher than that of those in the lecture group. |
| Baker et al., 2014 (88) | USA | Quantitative study | teachers & counselors | To implement and evaluate a culturally grounded school-based (Respect Curriculum) sexual violence prevention curriculum. | Findings provide preliminary support for the utility of a train-the-trainer model in addressing sensitive health topics. |
| Banegas et al., 2020 (89) | Argentina | Quantitative study | pre-service teachers | To analyze the impact of a foreign language teaching module with a gender perspective on the education of student teachers. | The student–teachers found the experience beneficial increased awareness of gender matters and their ability to produce motivating language activities anchored in wider social practices which promote gender equality and diversity. |
| Boozalis et al.,2020 (90) | Uganda | Quantitative study | teachers | To introduce and evaluate a comprehensive sex education curriculum to prevent pregnancies and sexually transmitted infections at a secondary school in Uganda. | There was a statistically significant improvement in student knowledge about pregnancy, contraceptives, and STIs. We also noted an increase in positive attitudes toward condom use. |
| Botfield et al., 2021 (91) | Fiji | Mixed methods study | teachers | To increase CSE access for students with disability to age-appropriate sexuality education. | At project completion, teachers reported increased confidence overall in teaching about sexuality and relationships. Interview findings suggested the project contributed to overcoming silence and stigma for people with disability and promoting safety for young people. |
| Browes, 2015 (49) | Ethiopia | Qualitative study | teachers | To understand the effects of the cultural setting, considering how gender and sexuality norms influence teacher and student implementation strategies. | Results show that CSE teachers and students, both male and female, were able to discuss issues of sexuality. However, the cultural context was seen to affect interpretation of program information, influencing the nature of this discussion. |
| Burns and Hendriks, 2018 (92) | Australia | Mixed methods study | teachers | To describe the formative, process and short-term impact evaluation of the two-day teacher training workshops designed to support the introduction of sexuality and relationships (SRE) curriculum delivered over a three-year period | Workshop evaluation found statistically significant improvements in attitudes towards SRE and increased comfort in teaching SRE, facilitating discussion and facilitating skills and activities, after the workshop. |
| Buston et al., 2002 (93) | Scottland | Mixed methods study | teachers | This paper focused on factors that impeded or facilitated the implementation of a specially designed sex education program, SHARE, which 13 Scottish schools were allocated to deliver in a randomized trial. | Implementation was hindered by competition for curriculum time, brevity of lessons, low priority accorded to PSE by senior management, particularly in relation to timetabling, and teachers' limited experience and ability in use of role-play. |
| Byrne et al., 2016 (94) | England | Quantitative study | pre-service teachers | To discuss a curriculum, change in the provision of health promotion in pre-service teacher education in a one-year postgraduate certificate in education secondary course in one Higher Education Institution in England. | The majority of the respondents found the training useful, felt confident and knowledgeable teaching and dealing with health issues, and held positive attitudes about promoting health. |
| Byrne et al., 2012 (95) | England | Mixed methods study | pre-service teachers | To report on Phase 1 of an 18-month project examining the long-term impact of an innovative health education program, based on socio-constructivist learning and critical reflection, during preservice teacher training. | The changes made to the health promotion component of the program and their implementation would not have been possible without the inter-professional collaboration that took place over three years. |
| Castillo-Nuñez et al., 2024 (96) | Ecuador | Qualitative study | teachers | To explore the experiences of teachers who work with adolescents across different subject areas with regard to the implementation of SE as a cross-curriculum subject. | The findings of this study show that, for teachers it is difficult to implement SE as a cross-curriculum approach axis of the school curriculum syllabus as stipulated by official laws. |
| Chavula et al., 2022 (97) | Zambia | Qualitative study | teachers | To contribute to the understanding of barriers and enabling factors for the implementation of CSE in a low/middle income country. | Training and providing a detailed teaching manual with participatory approaches for delivering CSE, and collaborative teaching enabled teachers and community health workers to easily communicate sensitive SRH topics to the learners. |
| Curtiss and Ebata, 2016 (98) | USA | Quantitative study | teachers | To examine a process to build capacity for individuals with autism to receive human sexuality education through training professionals via a 1-day workshop and providing ongoing education online. | In general, the climate among attendees was positive for teaching human sexuality education both in terms of their own values and their perceptions of support by others. The workshop and follow-up online education were effective for increasing instructional behavior and feelings of readiness. |
| Decker et al., 2022 (99) | USA | Quantitative study | teachers | To assess the perceptions and experiences of health educators regarding the integration of technology into a sexual health education program called In the Know and to identify facilitators and challenges to incorporating technology into the in-person curriculum. | The health educators generally had positive comments about the app and youth engagement with the technology-based content and activities; however, they also noted certain barriers to adolescents’ use of the mobile app including limited data storage and battery life on mobile phones. |
| Denny and Young, 2006 (100) | USA | Quantitative study | teachers | This article examined the results from an 18-month follow-up evaluation of the Sex Can Wait curriculum series. | Results indicated short-term effects for upper elementary and high school students and long-term (18 month) benefits for upper elementary and middle school students. |
| Dewhirst et al., 2014 (101) | England | Mixed methods study | pre-service teachers | To describe the practical implementation, content and evaluations, from 2012 and 2013, of a "Health Day" for pre-service teachers, including the effects on their confidence, knowledge, awareness and attitudes towards health and well-being/Personal Social Health and Economic Education. | Qualitative findings indicate that pre-service teachers at Southampton developed an understanding of the complexity of school health education and the roles of the health, education and voluntary sectors with respect to school health. It has also been noted that inter-sectoral and multi-agency networks are essential in developing and delivering this curriculum. |
| Dlamini et al., 2012 (102) | Nigeria | Mixed methods study | teachers & counselors | This paper presented the history and development of the Family Life and HIV Education (FLHE) program in Edo State, Nigeria and results of evaluation of teacher actions and responses to training in its delivery. | Results indicate that teachers benefited from the training, were aware of new and/or existing teaching resources and began to teach about HIV/AIDS. Teachers expressed that the program, facilitated open dialogue about HIV/AIDS. |
| Drake et al., 2015 (103) | USA | Quantitative study | teachers | To present the results from a randomized controlled trial to evaluate the effectiveness of the RTRworks! training intervention with respect to improving educators‚ implementation fidelity of the RTR program in middle and high school classroom settings. | The online training group reported significantly higher overall implementation fidelity compared to the standard control group with modest effect sizes and significant improvements in knowledge of the curriculum and perceptions of knowledge and skills in role-play improvement. |
| Eisenberg et al., 2010 (104) | USA | Qualitative study | teachers | To explore pre-service training experiences and needs of sexuality educators in Minnesota. | Results indicate a wide variety of pre-service teaching experience, ranging from no instruction to extensive training. |
| Ezer et al., 2022 (105) | Australia | Quantitative study | teachers | The study this article reports on the examined relationships between the type and amount of training of teachers nationally in Australia, and how it would impact their heir comfort teaching sexuality education around the release of the AC:HPE curriculum. | Teachers who had received any training or professional development had higher scores on having had the “right” training and spent more hours on delivery of sexuality education; 10–20 h or more of training was more strongly affirmed as useful. |
| Fasya et al., 2019 (106) | Indonesia | Mixed methods study | teachers | To improve the knowledge, attitude, and practice of teachers at eight schools for special needs children in Bandung City through the use of psychoeducation for teaching reproductive health to adolescents with intellectual disabilities. | This study demonstrates that there was an improvement in teacher knowledge and a change in teacher attitude from before and after a training intervention. |
| Gudyanga et al., 2019 (107) | Zimbabwe | Qualitative study | teachers and counselors | To explore how Guidance and Counseling teachers could be enabled to teach the necessary critical content in sexuality education in the HIV and AIDS education curriculum. | The participatory visual methodology, enabled a process in which the Guidance and Counseling teachers could reflect on themselves, the context in which they taught, their sexuality education work and learn how to navigate the contradictions and tensions. |
| Gursimsek, 2010 (108) | Turkey | Quantitative study | pre-service teachers | To see if teacher candidates’ attitudes towards sexuality and homosexuality change by attending a sexual education course for one semester. | Results demonstrate that sexuality education has a positive effect on participants' attitudes towards sexuality. In contrast, the course does not have any influence on candidates’ approach to homosexuality. |
| Hanass-Hancock et al., 2018 (109) | South Africa | Qualitative study | teachers, social workers, counselors, & psychologists | This paper focuses on the experience of educators who were trained with the 'Breaking the Silence' approach in its current form and here in particular on educators of learners with intellectual disabilities. | They revealed that the training and tools enabled them to provide sexuality education in accessible formats, tackle difficult topics such as sexual orientation and masturbation, and improved awareness and assertiveness within their learners. |
| Henderson et al., 2007 (110) | Scotland | Quantitative study | teachers | To assess the impact of a theoretically based sex education program (SHARE) delivered by teachers compared with conventional education in terms of conceptions and terminations registered by the NHS. | In an intention to treat" analysis there were no significant differences between the groups in registered conceptions per 1000 pupils and terminations per 1000 pupils between ages 16 and 20. |
| Housman et al., 2018 (111) | USA | Mixed methods study | teachers | To describe changes in participants perceptions and adoption of evidence-based sexuality education after attending a unique professional development opportunity for teachers of sexuality education using a Diffusion of Innovation Theory framework. | Statistically significant changes were found between pre- and post-test scores in relative advantage, compatibility, complexity, and observability. |
| Jarpe-Ratner and Marshall, 2021 (112) | USA | Mixed methods study | teachers | To understand how teachers approach the implementation of sexual health education (SHE), specifically in Chicago Public Schools and to understand what kinds of adaptations are being made and why teachers are making them. | Teachers made numerous adaptations, primarily aimed to respond to students’ needs and interests. |
| Johnson et al., 2014 (113) | Australia | Qualitative study | teachers | This study examines the implementation of a 10-lesson pilot relationships and sexuality education unit of work and an accompanying assessment task in two primary schools in South-East Queensland, Australia. | The results show the provision of a high-quality relationships and sexuality education curriculum resource grounded in contemporary educational principles and practices enables teachers to feel more confident to deliver relationships and sexuality education and minimizes potential barriers. |
| Keogh et al., 2021 (114) | Ghana, Kenya, Peru and Guatemala, | Mixed methods study | teachers | This multi-country mixed-methods study analyzed challenges to the implementation of national CSE curricula in schools in Ghana, Kenya, Peru and Guatemala, | Teaching methods lacked interactive activities necessary to develop skills and values. Increasing teacher training and distributing comprehensive CSE materials responsive to adolescents' needs remain priorities in all countries. |
| Kotkowski et al., 2022 (115) | Ecuador | Mixed methods study | teachers | To establish a pilot approach to comprehensive sex education facilitator training as applied in Riobamba, Ecuador. | Responses to anonymized surveys indicated improved self-perceived confidence in teaching each curriculum section. More objective pre- and post-teach-back evaluations showed improved ability to teach randomly assigned lessons as assessed by trainers. |
| LaChausse et al., 2014 (116) | not specified | Quantitative study | teachers | To examine how teacher characteristics affected program fidelity in an impact evaluation study of the Positive Prevention PLUS program, and to propose a comprehensive teacher training and professional development structure to increase program fidelity. | Compared with non-health credentialed teachers, credential health education teachers had greater comfort and self-efficacy regarding sex-related instruction. Teacher self-efficacy and comfort were significant predictors of adherence. |
| Lokanc-Diluzio et al., 2007 (117) | Canada | Mixed methods study | teachers | This article outlines the objectives, content, diverse methods of delivery, and evaluation of 11 such in-services for either elementary or junior high school teachers. | Pre- and post-surveys showed self-reported increases in knowledge, comfort and perceived ability to present accurate information. |
| Ma’rifah and Martha, 2019 (118) | Indonesia | Qualitative study | teachers | This study aimed to obtain extensive information on the sex education practices that have been carried out by teachers at the school for the disabled, Special School-C Dharma Asih, in Depok | The results showed that sex education was provided classically and incidentally at Special School-C Dharma Asih. The teachers also believed that sex education should be given to adolescents with mental retardation as early as possible but faced barriers. |
| Mahfuz et al., 2021 (119) | Bangladesh | Qualitative study | teachers and school nurses | To assess the perspectives of Bangladeshi teachers on the feasibility of delivery and potential for long-term sustainability of puberty and menstruation education in urban and rural schools | Participants found that the training and instructors’ manual they received were useful tools for effectively communicating with students. |
| Maia and Vilaça, 2020 (120) | Brazil | Qualitative study | teachers | To investigate teachers’ conceptions about sexuality and disabilities and the effects of an in-service training workshop in sexuality education. | The teachers came to view people with disabilities as sexual beings, clarified their beliefs, and planned an intervention project for their students with disabilities. |
| Mantell et al., 2006 (121) | South Africa | Qualitative study | teachers and school nurses | This study observed the impact of The Mpondombili Project which is a school-based intervention in rural KwaZulu-Natal that aims to promote delay in the onset of sexual activity and condom use as complementary strategies for both sexually experienced and inexperienced youth. Interactive training was carried out with peer educators, teachers and nurses over a 15-month period, and a manual developed. | The diversity of young people's relationships and vulnerability to sexual risk call for the promotion of both risk avoidance and risk reduction together, regardless of ideology, especially where HIV is well-established, to protect their health. |
| Martin et al.,2020 (122) | Iran | Quantitative study | teachers | The present study aimed to determine the effects of a preschool sex education program on preschool teachers’ knowledge and attitude. | Mean scores of knowledges and attitude in all dimensions showed a significant increase in the experimental group following the educational intervention. However, no difference was observed in the control group. |
| Maticka-Tyndale et al., 2014 (123) | Kenya | Quantitative study | teachers | This article addresses the question of replicability of outcomes of Primary School Action for Better Health. This article reports pre-10-month post- and 22-month post-training results as Primary School Action for Better Health was delivered in five additional regions of the country. | Results demonstrated positive gains in knowledge, self-efficacy related to changes in sexual behaviors and condom use, acceptance of HIV+ students, endorsement of HIV-testing and behaviors to post-pone sexual debut or decrease sexual activity. |
| McInnes and Ey, 2019 (124) | Australia | Qualitative study | teachers | This paper reports on the professional development, workplace and community support for educators and affected children, which educators and carers wanted to have implemented to more adequately respond to their professional needs and the needs of affected children and their families. | There is a clear and urgent need to act to reduce the prevalence and impacts of PSB to reduce the stress, distress and vicarious trauma which educators and carers are experiencing and to ensure that children are able to be safe in care and education sites |
| Morgan et al., 2004 (125) | England | Quantitative study | teachers | This paper reports the development of a radical approach to reward the achievements of peer educators working on a school-based sex and relationships education program. | Initial data suggest this has been extremely well received by teachers and peers. |
| Ocran, 2021 (126) | Ghana | Qualitative study | school based coordinators, head teachers, & a municipal school health education coordinator | This study aimed to investigate the training and support offered to teachers on the delivery of sex education in three contrasting Junior High Schools in Ghana, the attitudes and approaches to the delivery of sex education, and the response of students to teacher delivery of sex education. | The individual values and attitudes, as well as the type of school support, affects teacher modes of delivery and influences student interest in sex education. |
| Ollis, 2016 (127) | Australia | Qualitative study | teachers | This paper reports on data collected as part of a five-year project designed to implement a health promoting and whole school approach to sexuality education in a five-campus year 1-12 college in regional Victoria, Australia. | With adequate preparation, a framework to celebrate sex and sexuality, a gender lens to examine normative discourses, and the opportunity for reflection, teachers, can develop the confidence, skill and willingness to include pedagogies of pleasure in their school-based work. |
| Ollis and Harrison, 2016 (128) | Australia | Quantitative study | pre-service teachers | This paper explores the experience of teaching about pleasure to pre-service health and physical education teachers as part of compulsory studies in a unit on sexuality education designed to prepare them to teach sexuality education in secondary schools. | Teachers and key support staff have engaged in professional learning, a mentor program has been set up, a community engagement/parent liaison position has been created, and parent forums have been conducted on all five campuses. |
| Petersen et al., 2011 (129) | South Africa | Qualitative study | pre-service teachers | The article describes the use of a simulation game in HIV/AIDS education with pre-service teachers in Johannesburg, South Africa. | Content analysis and discourse analysis led to the construction of three main themes: the novelty factor of the simulation game for raising HIV/AIDS awareness was confirmed both during the game itself & after a period of time had elapsed, the game prompted more reflexivity about the disease and helped to broaden the participants’ discussions, & the data revealed the disjuncture between theory and practice in HIV/AIDS education. |
| Ram and Mohammadenzhad., 2020 (130) | Fiji | Qualitative study | teachers | This study aimed to explore the perceptions of teachers regarding the delivery of sexual and reproductive health education in secondary schools in Fiji. | Teachers viewed schools as ideal place for delivering sex education, however, could not consistently implement due to a lack of adequate information and skills, feared negative parental reaction, felt uncomfortable delivering sensitive topics and in most cases felt apprehensive to discuss sexuality issues in light of lack of training and resources. |
| Ramírez-Villalobos et al., 2021 (131) | Mexico | Quantitative study | teachers | The purpose of this article was to evaluate the knowledge of public secondary school teachers who received training in comprehensive education in sexuality and estimate the counseling effect on students’ sexual behavior. | Teachers increased their knowledge of sexuality after training. |
| Renju et al., 2010 (132) | Tanzania | Qualitative study | teachers | To present the results of a process evaluation examining the effect of a 10-fold scale-up of a school-based adolescent sexual and reproductive health intervention on teacher attitudes and experiences in Mwanza Region, Tanzania. | The training was well implemented and led to some key improvements in teachers' adolescent sexual and reproductive health knowledge, attitudes and perceived self-efficacy, with substantial improvements in knowledge about reproductive biology and attitudes towards confidentiality. |
| Rijsdijk et al., 2014 (133) | Uganda | Mixed methods study | teachers | The purpose of this mixed-methods study was to examine factors associated with dose delivered (number of lessons implemented) and fidelity of implementation (implementation according to the manual), as well as to identify the main barriers and facilitators of implementation of the World Starts With Me program. | Confidence in educating and discussing sexuality issues in class was positively associated with fidelity of implementation, whereas the importance teachers attached to open sex education showed a negative association with fidelity. Main barriers for implementing World Starts With Me were lack of time, unavailability of computers, lack of student manuals and lack of financial support and rewards. |
| Rose et al., 2019 (134) | USA | Qualitative study | teachers | This study had two primary aims:(a) identify factors that diminish or enhance middle school health education teachers comfort implementing sexual health education, (b) identify factors that diminish or enhance middle school students’ receipt of this education. | Findings identified key barriers including disruptive behavior, insufficient time, and lack of dedicated classrooms. Some key facilitators to comfort included professional development and establishing ground rules. |
| Schutte et al., 2018 (135) | The Netherlands | Quantitative study | teachers | This study aimed to report on the effect evaluation to determine the effect of the Web-based e-coach on teacher implementation of a school-based sex education program called Long Live Love and on its determinants. | The e-coaching intervention was not found to have an effect on teachers’ implementation behavior; teachers assigned to the experimental e-coaching website did not score higher on completeness as compared with teachers in the control condition. |
| Sediyono et al., 2018 (136) | Indonesia | Qualitative study | teachers & principals | This study developed a multimedia-based teaching media, animation, and user-friendly digital books to empower these teachers. The other aim to be achieved was the formation of communication and parent-student-teacher partnership. | The impact of this action program was the establishment of tranquility and comfort in the community's social life. The teachers were increasingly skilled and encouraged to have courage in socializing anti-sexual violence to the students. |
| Sinkinson, 2009 (137) | New Zealand | Mixed methods study | pre-service teachers | This paper presents the findings of a three-year study into pre-service (student) teachers' experiences of and beliefs about sexuality education in New Zealand schools. It reports on participants' own memories of school sexuality education programs and examines changes in their constructs of sexuality education during their teacher education in health education. | By the end of 2006, although physical safety and avoiding risk were still presented as important aspects of sexuality education, a theme of 'sexuality isn't just about sex' had emerged. Positive and comprehensive approaches to teaching about sexuality now defined their constructs of sexuality education. |
| Smith et al., 2013 (138) | South Africa | Qualitative study | teachers | This study investigated the attitudes of 43 teachers and school administrators towards sex education, young people's sexuality and their communities in 19 secondary schools in rural KwaZulu-Natal, South Africa, and how these attitudes affect school-based HIV prevention and sex education. | Teachers perceived themselves to have higher personal standards and moral authority than members of the communities and schools they served. Male administrators' authority to determine school policies and teachers' attitudes towards sexuality fundamentally affect the content and delivery of school-based sexuality education and HIV prevention activities. |
| Thammaraksa et al., 2014 (139) | Thailand | Quantitative study | teachers | The purpose of this quasi-experimental study was to evaluate the effects of Culturally Sensitive Sex Education Skill Development, a teacher-led sex education program in secondary schools in Thailand. The program aimed to enhance teachers' skills in communicating with students’ information on sexual issues especially those never addressed in a formal curriculum (i.e., sexual communication, awareness about sexual health barriers, masturbation, and sexual imagination). | Primary outcomes were attitudes toward sex education, perceived self-efficacy, and sex education skills. Statistical analysis included independent and paired t test, and repeated one-way analysis of variance. |
| Van Lieshout et al., 2017 (140) | Netherlands | Qualitative study | teachers | The present study investigated the Long Live Love+ (LLL+) completeness and fidelity, factors influencing implementation, and student response. | Fidelity was high, but many teachers added elements. The most important factors that influenced implementation were time and organizational constraints, lack of awareness on the impact of completeness and fidelity, and student response. |
| van Reeuwijk et al., 2023 (30) | Indonesia | Qualitative study | teachers | This study examines key factors that facilitated and hindered creating an enabling environment for the implementation of SEmangaT duniA RemajA/Teens Aspirations (SETARA), a comprehensive sexuality education intervention in schools across three sites in Indonesia. | Findings showed that the relationship between the implementing organization and city government officials was important for obtaining approval, support, and formal agreements on collaboration. Framing the curriculum within local policies and priorities made it easier to communicate to schools, community, and parents. |
| Vilaça, 2011 (141) | Portugal | Qualitative study | teachers | This research describes the contributions for teachers‚ professional development of two different in-service teacher training modalities, a Workshop and a Training Course, whose principal aim was to prepare teachers to carry out action-oriented sexuality education projects in their schools. | A larger number of teachers who attended the Workshop than those involved in the Training Course, developed reflexive actions which led to positive changes in their methodological approaches of sexuality education and increased more student participation. |
| Vilaça, 2017 (142) | Brazil | Qualitative study | teachers | To discuss teachers’ practices, barriers and facilitating factors associated with a regional school-based action-oriented sexuality education (SE) project with the use of information and communication technology. | It was found that when teachers attend in-service teacher education and training, they develop professional competences to act as facilitators of students’ inquiry-based learning on action-oriented knowledge and on carrying out collective actions to promote sexual health and well-being. |
| Vilaça et al., 2011 (143) | Portugal | Qualitative study | teachers | To investigate how teachers', conceptions and practices change during in--service teacher training so as to create adequate conditions in the school and implement a sexual education project based on the Democratic Health Education Paradigm with the use of information and communication technology. | Considering its participative dimension, the results of this investigation have implications in terms of teacher training and the organization and management of the curricula. |
| Visser, 2005 (144) | South Africa | Mixed methods study | teachers | The aim of the research was to monitor the implementation of the life skills and HIV/AIDS education program in schools and to evaluate the impact of the intervention in terms of the knowledge, attitudes and reported behavior patterns of learners. | In an outcome evaluation over the period of a year it was found that learners' knowledge of HIV / AIDS increased, and their attitudes were more positive although the changes may not be attributed to the program alone. |
| Walker et al., 2003 (145) | UK | Mixed methods study | school nurses, senior management, a governor, & pastoral staff | To evaluate a sexual health training program designed to prepare school teams to deliver high quality SRE. | The training program was found to have made positive contributions to SRE program and policy development through building schools' capacity to be responsive to young people's needs, team building, personal and professional development, and greater collaboration in school between health and education services, parents and pupils. |
| Wang et al., 2022 (146) | The Bahamas | Quantitative study | teachers | This study investigated the implementation of an evidence-based intervention in collaboration with The Bahamas Ministries of Education and Health by comparing data from standard and enhanced implementation trials, which allows assessment of the relative contribution of an enhanced implementation approach to both implementation fidelity and program outcomes. | BMF and SAM were significantly associated with teachers’ implementation fidelity. Teachers who received both BFM and SAM taught the greatest numbers of core activities, followed by teachers who received either BMF or SAM. Teachers who did not receive BMF or SAM taught the lowest numbers. |
| Wang et al., 2022 (147) | The Bahamas | Quantitative study | teachers | To develop and refine two theory-driven implementation strategies (BMF and SAM) which demonstrated significant effects on teachers' fidelity of implementation of an evidence-based HIV intervention in school setting. | Teachers in the 2019–2020 trial taught more core activities & sessions than those participating in the 2011–2012 trial. Teachers who had a “very good” or “excellent” school coordinator in their schools taught more core activities than those who had a “satisfactory” school coordinator. Teachers’ confidence in implementing core activities, comfort level with the curriculum, attitudes towards sex education in schools, and perceived principal support were significantly related to increased self-efficacy, which in turn was related to teachers’ implementation fidelity. |
| Weingarten et al., 2018 (148) | USA | Mixed methods study | teachers, counselors, social workers, & behavioral health specialists | To analyze the utility of a train-the-trainer model of dissemination for a sexual violence prevention program in the state of Hawaii. | Time spent in training, job position, and time in that position predicted whether a person trained to implement the sexual violence prevention program followed through with teaching the program to students. |
| Wiefferink et al., 2005 (149) | The Netherlands | Mixed methods study | teachers | To focus on the determinants of teachers’ implementation of the revised Long Live Love curriculum, and on the effects of the applied innovation strategy on their classroom implementation and on related determinants. | The innovation strategy had a positive impact not only on extent of use, but also on teachers' curriculum-related beliefs. |
| Wight and Buston, 2003 (18) | Scotland | Qualitative study | teachers | This paper evaluates the teacher training component of a theoretically based behavioral sex education program for 13–15-year-olds called SHARE. | The two main objectives of the training program that were congruent with teachers' perceived needs were fulfilled: to make teachers more comfortable and confident to deliver sex education and to prepare them to deliver the SHARE pack. |
| Wight et al., 2002 (150) | Scotland | Quantitative study | teachers | To determine whether a theoretically based sex education program for adolescents (SHARE) delivered by teachers reduced unsafe sexual intercourse compared with current practice. | When the intervention group was compared with the conventional sex education group in an intention to treat analysis there were no differences in sexual activity or sexual risk taking by the age of 16 years. |
| Wilson et al., 2015 (151) | USA | Qualitative study | teachers | This article presents a framework for planning and implementing a professional development opportunity for public school teachers occurring outside the school setting. | The Sexuality Education Academy approach holds promise for professional development efforts in sexuality education, in addition to evaluating interventions to ensure accurate implementation of evidence-based sexuality education. |
| Wood, 2009 (152) | South Africa | Qualitative study | teachers | This article describes how South African educators were facilitated to adopt the role of HIV/AIDS‚ "ambassadors", within their circles of influence by participating in a two-year course for an Advanced Certificate in Education for HIV/AIDS in teaching qualification. | Based on the Freirian notion of liberation pedagogy, the article puts forward the argument that by adopting a holistic and critical approach to HIV/AIDS education, educators are able to move beyond the traditional responsibilities of the teacher, and thus play an important role as agents of change in their school and community. |
| Wood et al., 2015 (153) | Nigeria | Qualitative study | teachers | In the development of the national school-based HIV prevention curriculum in Nigeria, several state governments partnered with feminist (or like-minded) non-governmental organizations to collaborate on teacher training. This case study, drawing on teacher interviews and classroom observations, explores the effects of that experience. | Teachers reported that the 10-day training developed their competence, confidence, and commitment to foster students' critical thinking about gender issues. Specifically, they reported changes in their own gender attitudes, pedagogic skills and connectedness with students, particularly girls. |
| Yang, 2014 (154) | Taiwan | Qualitative study | pre-service teachers | The aim of this study is to present an analytical framework for developing students' sexual literacy through popular culture, while addressing the challenges teachers face in critically analyzing its ideology and creating motivating activities. | Through this framework, the true needs of students in sexuality education can be addressed. This pedagogical approach also relates the course content to the practical experiences of young students and alters student opinions on formal sexual education. |
